# Anti-cryptococcal activity of ethanol crude extract and hexane fraction from *Ocimum basilicum* var. Maria bonita: mechanisms of action and synergism with amphotericin B and *Ocimum basilicum* essential oil

**DOI:** 10.1080/13880209.2017.1302483

**Published:** 2017-03-19

**Authors:** Nathalia N. R. Cardoso, Celuta S. Alviano, Arie F. Blank, Maria de Fátima Arrigoni-Blank, Maria Teresa V. Romanos, Marcel M. L. Cunha, Antonio Jorge R. da Silva, Daniela S. Alviano

**Affiliations:** a Department of General Microbiology, Institute of Microbiology Paulo de Góes, Federal University of Rio de Janeiro, Rio de Janeiro, Brazil;; b Postgraduate Program in Plant Biotechnology, Federal University of Rio de Janeiro, Rio de Janeiro, Brazil;; c Department of Agronomy, Federal University of Sergipe, São Cristóvão, Brazil;; d Research Institute of Natural Products, Federal University of Rio de Janeiro, Rio de Janeiro, Brazil

**Keywords:** Basil, antifungal activity, *Cryptococcus neoformans*, drug synergism, ergosterol

## Abstract

**Context:***Ocimum basilicum* L. (Lamiaceae) has been used in folk medicine to treat headaches, kidney disorders, and intestinal worms.

**Objective:** This study evaluates the anti-cryptococcal activity of ethanol crude extract and hexane fraction obtained from *O. basilicum* var. Maria Bonita leaves.

**Materials and methods:** The MIC values for *Cryptococcus* sp. were obtained according to Clinical and Laboratory Standards Institute in a range of 0.3–2500 μg/mL. The checkerboard assay evaluated the association of the substances tested (in a range of 0.099–2500 μg/mL) with amphotericin B and *O. basilicum* essential oil for 48 h. The ethanol extract, hexane fraction and associations in a range of 0.3–2500 μg/mL were tested for pigmentation inhibition after 7 days of treatment. The inhibition of ergosterol synthesis and reduction of capsule size were evaluated after the treatment with ethanol extract (312 μg/mL), hexane fraction (78 μg/mL) and the combinations of essential oil + ethanol extract (78 μg/mL + 19.5 μg/mL, respectively) and essential oil + hexane fraction (39.36 μg/mL + 10 μg/mL, respectively) for 24 and 48 h, respectively.

**Results:** The hexane fraction presented better results than the ethanol extract, with a low MIC (156 μg/mL against *C. neoformans* T_444_ and 312 μg/mL against *C. neoformans* H99 serotype A and *C. gattii* WM779 serotype C). The combination of the ethanol extract and hexane fraction with amphotericin B and essential oil enhanced their antifungal activity, reducing the concentration of each substance needed to kill 100% of the inoculum. The substances tested were able to reduce the pigmentation, capsule size and ergosterol synthesis, which suggest they have important mechanisms of action.

**Conclusions:** These results provide further support for the use of ethanol extracts of *O. basilicum* as a potential source of antifungal agents.

## Introduction

Cryptococcosis is a fungal disease caused by *Cryptococcus* sp. that affects the central nervous system and has high levels of mortality. *C. neoformans* has several virulence factors such as the production of melanin and mannitol, capsular polysaccharide and sialic acids. One of the most important virulence factors of *C. neoformans* is the capsule that has anti-phagocytic properties and causes adverse effects on the host immune system (Doering 2009; Zaragoza et al. 2009).

In general, natural products, such as basil, are widely available and usually have a low cost. These plant extracts are commonly used in traditional medicine worldwide (Gurib-Fakim 2006; Zielińska & Matkowski 2014). A large number of studies have attributed different biological activities to natural products such as antiviral, antigiardial, antispasmodic, carminative, analgesic, healing, expectorant, antiseptic, respiratory tract and anti-inflammatory activities (Bruneton 1999; Jones et al. 2000; Melo et al. 2005; Ríos & Recio 2005; Lopes-Lutz et al. 2008).

*Ocimum basilicum* L. (Lamiaceae), popularly known as basil, is found in tropical and subtropical regions of Asia, Africa, Central America and South America (Zakaria et al. 2008). It is a medicinal herb widely used in folk medicine to combat headaches, coughs, intestinal worms, kidney disorders and as an antispasmodic agent (Lee et al. 2005). It is also used as a flavouring agent in sausages, meats, on pizzas and in salads. In addition, the essential oil (E.O.) is used in the cosmetic and perfumery industry. The antimicrobial activity of basil extracts and E.O. has been reported to be associated with its major and minor components, such as eugenol, linalool, methyl cinnamate, and methyl chavicol (Kocić-Tanackov et al. 2012).

Despite the many antifungal therapies available, cryptococcal infections show high mortality rates, especially in immunocompromised individuals. Besides, such therapies have many unpleasant collateral effects. Therefore, it is important to discover new antifungal agents derived from natural products. The aim of this study was to investigate the anti**-**cryptococcal activity of the ethanol crude extract (E.C.E.) and the hexane fraction (H.F.) from a genetically improved *O. basilicum* cultivar (*O. basilicum* var. Maria Bonita) against the *C. neoformans* strain.

## Materials and methods

### Chemicals

Amphotericin B (AMB) and resazurin were obtained from Sigma-Aldrich (USA) and stored according to the supplier’s instructions. All solvents used were of spectroscopic grade from Tedia (Fairfield, OH).

### Plant material

*Ocimum basilicum* L. var. Maria Bonita is a genetically improved cultivar, derived from the Germplasm Bank North Central Regional PI Station, USA (accession PI 197442). *O. basilicum* leaves were collected from Federal University of Sergipe in January and March 2013, where a voucher specimen was deposited (register number 13162).

### Preparation of plant extract

Absolute ethanol was used as the solvent to prepare the plant extract. Dried *O. basilicum* leaves (1.1 kg) were kept for 10 days in 13 L of ethanol and then filtered through Whatman No. 1 filter paper. The E.C.E. was suspended in distilled water and fractioned in a polarity scale with *n*-hexane, dichloromethane, ethyl acetate and *n*-butanol.

### GC/MS

GC/MS analyses of the active areas of H.F. observed in bioautography were performed in a Shimadzu QP2010 gas chromatography coupled to a Shimadzu QP2010 plus mass selective detector using the following conditions: column DB25 (30 m × 0.25 mm, film thickness 0.25 μm); helium was used as the carrier gas at a flow rate 1 mL/min; the column temperature was programed from 60 to 290 at 10 °C/min; and injector temperature was kept at 270 °C.

### Microorganisms and cell line

The *C. neoformans* T**-**444 strains were provided by Universidade Federal de São Paulo (UNIFESP). The *C. neoformans* H99 serotype A and *C. gattii* WM779 serotype C were kindly provided by prof. Dr. Márcio Lourenço Rodrigues. The microorganisms were maintained in Sabouraud dextrose agar for 48 h at room temperature.

The macrophage cell line RAW 264.7 was purchased from the Rio de Janeiro cell bank. Cells were grown in Dulbecco’s Modified Eagle Medium (DMEM) supplemented with 2 mM l-glutamine, 50 μg/mL gentamicin, 2.5 μg/mL fungizone, plus 10% of heat-inactivated foetal bovine serum (FBS) and maintained at 37 °C in a 5% CO_2_ atmosphere.

### Evaluation of minimum inhibitory concentrations

The minimum inhibitory concentration (MIC) was determined using the microdilution broth method according to CLSI M27-A (CLSI 2008). First, the *O. basilicum* E.C.E. and H.F. were serially diluted in 96-well plates in duplicates. Then 100 μL of cell suspension (10^3^ yeast/mL) was added to each well and the plate was incubated at room temperature for 48 h. Positive controls were prepared using yeast inoculated growth medium (untreated cells). Pure medium was used for the negative controls. Growth inhibition was confirmed after the addition of 30 μL of resazurin solution (5 mg/100 mL of phosphate buffer saline, PBS, pH 7.2) and further incubation at 37 °C for 3 h. AMB was used as the antimicrobial standard drug. MIC was defined as the lowest concentration that completely invalidated the microorganism growth.

### Synergism assay with the antifungal standard drug AMB

The synergistic effects of E.C.E., H.E. and E.O. on AMB antifungal activity were performed as previously described by Zore et al. (2011), with slight modifications. AMB and *O. basilicum* E.C.E. or H.E. were combined at concentrations lower than their individual MIC values in 96-well microplates. Each plate was inoculated with 10^3^ cells/mL and incubated at room temperature for 48 h. The results were based on visual growth, which were confirmed with the addition of resazurin as described above. Fractional inhibitory concentrations (FICs) for each substance and their combinations with AMB were calculated as follows: FIC*_x_* = concentration that inhibits 100% of growth in combination/concentration that inhibits 100% of growth alone.

The FIC*_index_*was calculated by adding both FIC values. The interaction was classified as synergistic when FIC*_index_* values <0.5, additive when 0.5 < FIC*_index_* ≤ 1; indifferent when 1 < FIC*_index_* > 4 and antagonistic when FIC*_index_* >4.0 (Schelz et al. 2006).

### Sorbitol assay

The sorbitol assay was determined using the microdilution broth method in the presence and absence of sorbitol solution (0.8 M), which acts as an osmoprotectant. If E.C.E. or H.F. acts in the cell wall, MIC values obtained with sorbitol will be higher than the MIC values without sorbitol after incubation. MIC was defined as the lowest concentration that completely invalidated the microorganism growth after seven days of incubation (Frost et al. 1995).

### Kill-curve

The kinetics of the anti-cryptococcal activity of E.C.E. and H.F. were studied by the time-dependent kill-curve assay using *C. neoformans* T_444_ strain. Sabouraud broth (200 μL) containing 2.5 × 10^3^ CFU/mL of *C. neoformans* and minimum fungicidal concentrations (MFC) of E.C.E. and H.F. were incubated. Aliquots of 20 μL were taken at different intervals of time, and then re-suspended in 180 μL sterile saline and inoculated on Sabouraud agar plates. Plates were incubated at 37 °C for 48 h. The number of colonies was counted and compared with control (without test compound).

### Ergosterol quantification

The amount of ergosterol was determined according to Arthington-Skaggs et al. (1999), with a slight modification. A single *C. neoformans* colony from an overnight Sabouraud dextrose agar plate culture was inoculate in 50 mL of Sabouraud dextrose broth containing different concentrations of the E.C.E. (312 μg/mL), H.F. (78 μg/mL), E.C.E. and E.O. (19.5 and 78 μg/mL, respectively) and H.F. and E.O. (10 and 39.36 μg/mL, respectively). *C. neoformans* cultures were incubated at 37 °C for 24 h with shaking. The cells were harvested by centrifugation and washed with sterile water. The wet weight of the cell pellet was determined. Three milliliters of 25% alcoholic potassium hydroxide solution (25 g of KOH and 36 mL of sterile distilled water, brought to 100 mL with 100% ethanol) was added and mixed by vortex for 1 min. Cell suspensions were incubated in a water bath for 1 h at 85 °C. After incubation, the tubes were cooled to room temperature. The sterols were extracted by the addition of 1 mL of sterile distilled water and 3 mL of cyclohexane, and then mixing by vortex for 3 min. The cyclohexane layer was transferred to a clean borosilicate glass tube. A 200 μL aliquot of the sterol extract was diluted fivefold in 100% ethanol and scanned spectrophotometrically between 200 and 300 nm (DU 530 Life Science UV/Visible Spectrophotometer, Beckman Coulter) (Arthington-Skaggs et al. 1999).

Both 24(28)-dehydroergosterol (DHE), a late sterol pathway intermediate, and ergosterol absorb at 281.5 nm, but at 230 nm only 24(28) DHE shows an absorption spectrum. Ergosterol content was calculated as a percentage of the wet weight of the cells using the following equations: % ergosterol + % 24(28) DHE = [(*A*_281.5_/290) × *F*]/pellet weight; % 24(28) DHE = [(*A*_230_/518) × *F*]/pellet weight, and % ergosterol = [% ergosterol + % 24(28) DHE] − % 24(28) DHE, where *F* is the factor for dilution in ethanol and 290 and 518 are the *E* values determined for crystalline ergosterol and 24(28) DHE, respectively (Breivik & Owades 1957).

### Effect of E.C.E. and H.F. and its association with E.O. On pigmentation

The evaluation of the effect of the E.C.E, H.F. and its association with E.O. on pigmentation of *C. neoformans* was performed using the minimum broth medium supplemented with 1 mM L-DOPA (L-3,4-dihydroxyphenylalanine) to induce pigmentation. The E.C.E. and H.F. were diluted according to the CLSI reference document for yeast. Different concentrations were obtained, and then incubated in a dark humid chamber at 37 °C for 7 days. Minimum broth medium without L-DOPA was used as the negative control of pigmentation. The results were obtained with a spectrophotometer in wavelength at 400 nm (Turick et al. 2002).

### *C. neoformans* capsule size

*Cryptococcus neoformans* was incubated for 48 h at 35 °C in the presence of sub-inhibitory concentrations of the E.C.E. (312 μg/mL), H.F. (78 μg/mL), E.C.E. and E.O. (19.5 and 78 μg/mL, respectively) and H.F. and E.O. (10 and 39.36 μg/mL, respectively). After 48 h, an aliquot was removed and fixed with a formaldehyde solution (10% formaldehyde in PBS) to measure capsule sizes. Slides were prepared for measurements with a dilution of 1:3 of cells and India ink dye to enable capsule visualization. The images were obtained using a Zeiss Axioplan microscope. Measurements were performed using ImageJ (Abramoff et al. 2004).

### Cytotoxicity

E.C.E. and H.F. were suspended in 1% dimethyl sulfoxide (DMSO), and stock solutions were prepared in water at 2000 μg/mL and sterilized by filtration using a 0.22 μm Millipore membrane filter. The cytotoxicity assay was performed by incubating RAW cell monolayers with two-fold serial dilutions of E.C.E and H.F. for 48 h at 37 °C in 5% CO_2_ atmosphere. Cellular viability was further evaluated by the neutral red dye-uptake method (Borenfreund & Puerner 1985).

## Results and discussion

**Figure 1.**
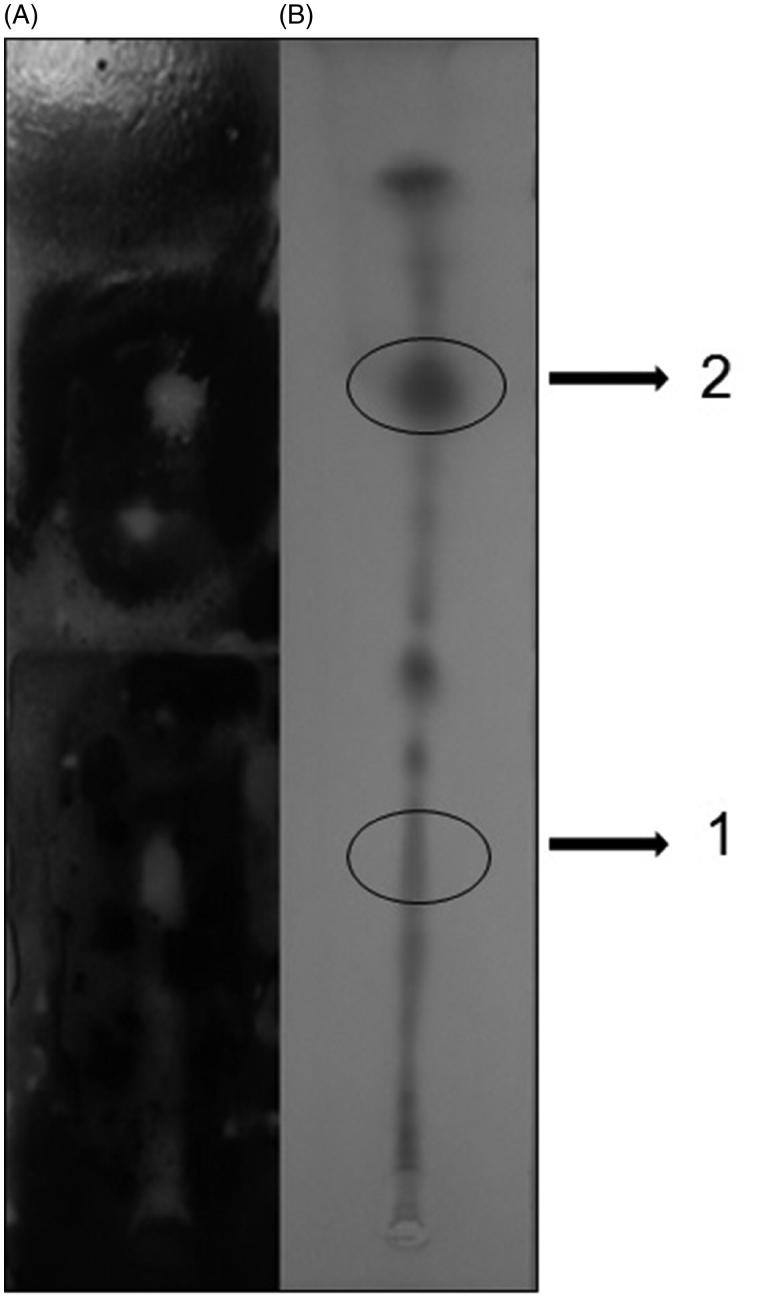
Bioautography technique and thin layer chromatography (TLC): 2 μL H.F. and solvent system (hexane and ethyl acetate, 75:25). (A) Bioautography and (B) TLC.

The results of minimum inhibitory concentrations are shown in Table 1. The MIC values found for the H.F. against the three *Cryptococcus* strains were lower than the E.C.E. H.F. and E.C.E. were more effective against *C. neoformans* T_444_ (156 and 625 μg/mL, respectively). H.F. and E.C.E. presented MIC values of 312 and 1250 μg/mL, against *C. neoformans* H99, respectively. The MIC values for H.F and E.C.E. were 312 and 2500 μg/mL against *C. gattii* WM779, respectively. These results corroborate with reports in the literature that show antimicrobial properties of extracts and essential oils (Falcão et al. 2008; Fabri et al. 2012; Silva-Belmares et al. 2014; Chaftar et al. 2015; Dezaki et al. 2015; Marinas et al. 2015; Satyal et al. 2015; Cardoso et al. 2016). As the best results were obtained with *C. neoformans* T_444_, this strain was chosen for the following experiments. As the H.F. presented better results, it was submitted to the bioautography technique. This assay showed two distinct areas of activity (Figure 1) that were analyzed by GC/MS.

**Table 1.** Minimum inhibitory concentration of E.C.E. and H.F. against *Cryptococcus* sp.

	*Cryptococcus neoformans* T_444_	*Cryptococcus neoformans* H99	*Cryptococcus gattii* WM779
MIC (μg/ml)			
E.C.E.	625	1250	2500
H.F.	156	312	312
E.O.	1250	–	–

E.C.E.: ethanol crude extract; H.F.: hexane fraction; E.O.: essential oil.

**Figure 2.**
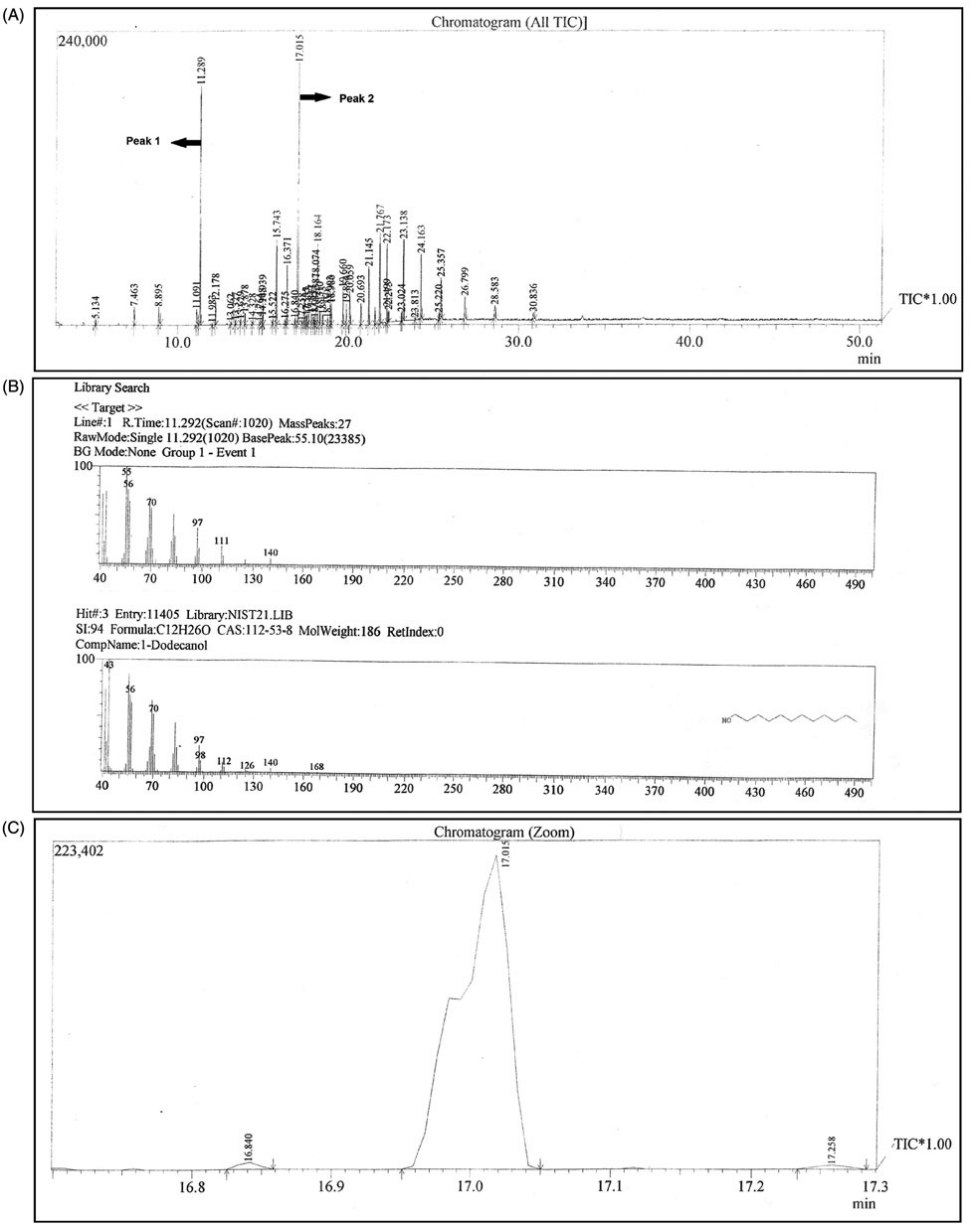
Gas chromatography coupled to mass spectrometry (GC-MS): (A) chromatogram, (B) mass spectrum of identified component *–* peak 1 and (C) amplified chromatogram of peak 2.

The identification of the two areas shows some substances that are probably responsible for the antimicrobial activity found. Area 1 shows a complex mixture with two major peaks. The first peak (RT 11.289 min) was identified as 1-dodecanol (with a similarity index of 94%), while the analysis of the second peak (RT 17.015 min) was inconclusive, as there was a mixture of two substance (Figure 2(A–C)). Togashi et al. (2007) showed the antibacterial activity of 1-dodecanol against *Staphylococcus aureus* (Togashi et al. 2007). Hall et al. showed that 1-dodecanol regulates morphogenesis in *Candida albicans* (Hall et al. 2011).

**Figure 3.**
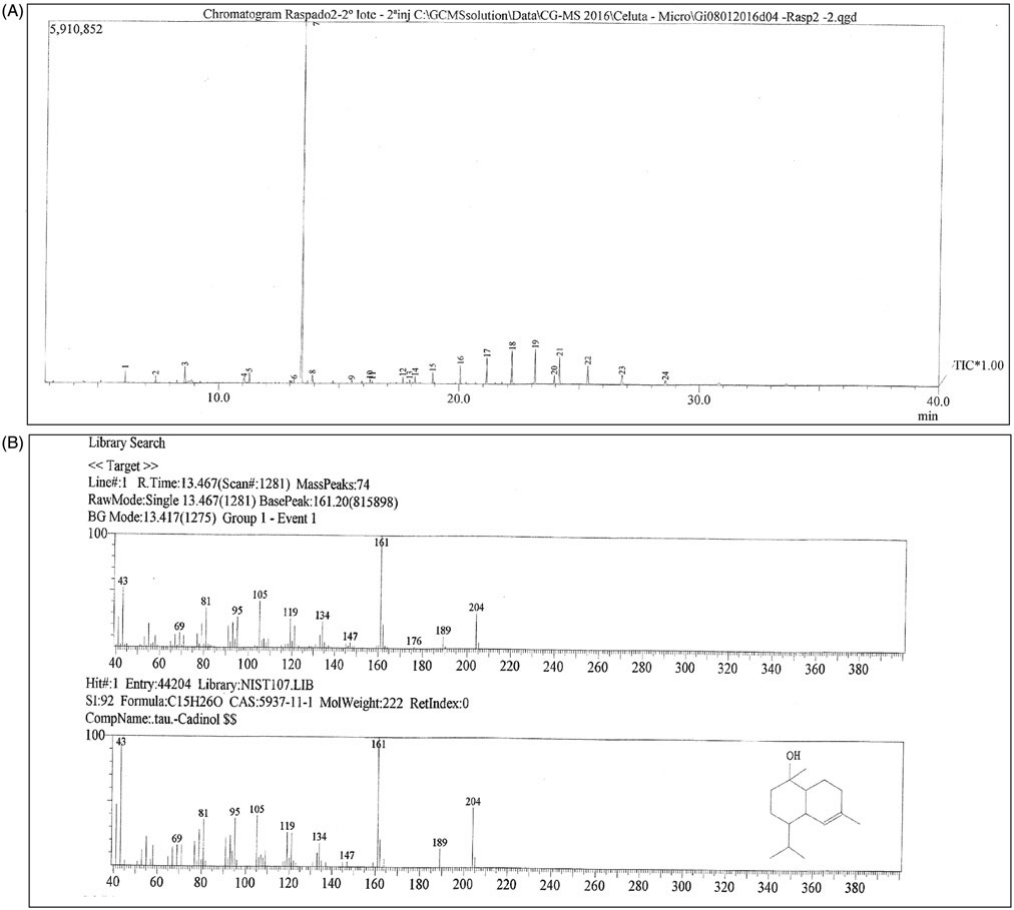
Gas chromatography coupled to mass spectrometry (GC-MS): (A) chromatogram and (B) mass spectrum of identified component.

The analysis of area 2 identified T-cadinol as being responsible for the activity found with a similarity index of 92% (Figure 3(A,B)). T-cadinol is a sesquiterpene and the presence of T-cadinol has been reported in some essential oils (Maia et al. 2005; Tung et al. 2008; Mothana et al. 2010; Özek et al. 2014). Parnema et al. (2012) suggested that the activity against *Escherichia coli* and *Staphylococcus aureus* was due to the presence of T-cadinol in the dichloromethane extract of *Monanthotaxis discolor* (Parnema et al. 2012). Boussaada et al. (2008) reported the presence of T-cadinol in the active volatile fractions of *Rhaponticum acaule* against *Staphylococcus aureus*, *Staphylococcus epidermidis* and *Salmonella typhimurium* (Boussaada et al. 2008).

The presence of T-cadinol in the hexane fraction of *O. basilicum* in this study corroborates with the study of Rosas (2005) that found T-cadinol in the hexane and dichloromethane extracts of *Raputia praetermissa* (Rosas, 2005). Zapf et al. (1996) reported a low cytotoxicity of T-cadinol in HeLaS3 and BHK-21 cells, with IC_50_ ranging between 25 and 50 μg/mL (Zapf et al. 1996). The presence of T-cadinol in *O. basilicum* ethanol extracts was not found in the consulted literature.

Next, we evaluate the combination of E.C.E., H.F. with the standard drug, AMB and their combinations with the E.O. of the same plant. The MIC value from E.O. was obtained from a previous study by our group (Cardoso et al. 2016). Some work has reported the combination of different antifungal agents or of natural products and standard drugs in reducing their MIC values (Faria et al. 2011; Liu et al. 2014). In this work, all the combinations tested produced FIC_*index*_ values ranging from 0.187 to 0.75. This showed that all these combinations reduced the MIC values. The synergistic effect was observed in the combination of AMB and E.C.E., reducing their MIC from 1.56 to 0.099 μg/mL and 625 to 78 μg/mL, respectively; in the combination of E.C.E. with E.O., reducing their MIC values from 625 to 39 μg/mL and 1250 to 157.2 μg/mL, respectively, and in the combination of H.F. and E.O., reducing their MIC from 156 to 20 and 1250 to 78.72 μg/mL, respectively. When AMB was combined with 78 μg/mL H.F., their MIC values were reduced from 1.56 to 0.396 (Table 2).

**Table 2.** Evaluation of interaction resulting from the combination of the substances tested by determining the FIC*_index_* using the checkerboard technique.

	*Cryptococcus neoformans*	T_444_
MIC (μg/mL)	E.C.E.	625
	H.F.	156
	E.O.	1250
	AMB	1.56
MIC in combination (μg/mL)	E.C.E.	39
	E.O.	157.2
	FIC index	0.187 (S)
	H.F.	20
	E.O.	78.72
	FIC index	0.19 (S)
	E.C.E.	78
	AMB	0.099
	FIC index	0.188 (S)
	H.F.	78
	AMB	0.396
	FIC index	0.75 (A)

E.C.E.: ethanol crude extract; H.F.: hexane fraction; E.O.: essential oil; AMB: amphotericin B; FIC index: fractional inhibitory concentration index; (S): synergistic; (A): additive.

The association between the E.C.E and H.F. with *O. basilicum* E.O. potentiated the activities of the three substances separately. The synergistic effect was observed in the association of E.C.E. and E.O. with a MIC reduction of 16× and 8×, respectively. The synergistic effect was observed in the association of H.F. with E.O. (reduction of 8× and 16×, respectively) and in the combination of AMB with E.C.E. (reduction of 16× and 8×, respectively). Only the combination of AMB with H.F. result in an additive effect, where both had their MIC reduced by 4× and 2× in the association, but the value of FIC_*index*_ was higher than 0.5.

The fungal cell wall acts as a barrier, conferring rigidity and strength. It is composed of several macromolecules essential for fungal survival like β-glucan, chitin and other proteins and it is an important target for antifungal agents (Lee & Kim 2016).

We evaluated the mechanism of action of the E.C.E. and H.F. on the cell wall of *C. neoformans* by the addition of an osmoprotectant. The addition of sorbitol to the culture medium did not affect the MIC value of the E.C.E., even after 7 days of growth, showing that it has no effect on the fungal cell wall. The treatment with H.F. resulted in an increase in the MIC value when treated in the presence of sorbitol, indicating that the H.F. affects the cell wall.

**Figure 4.**
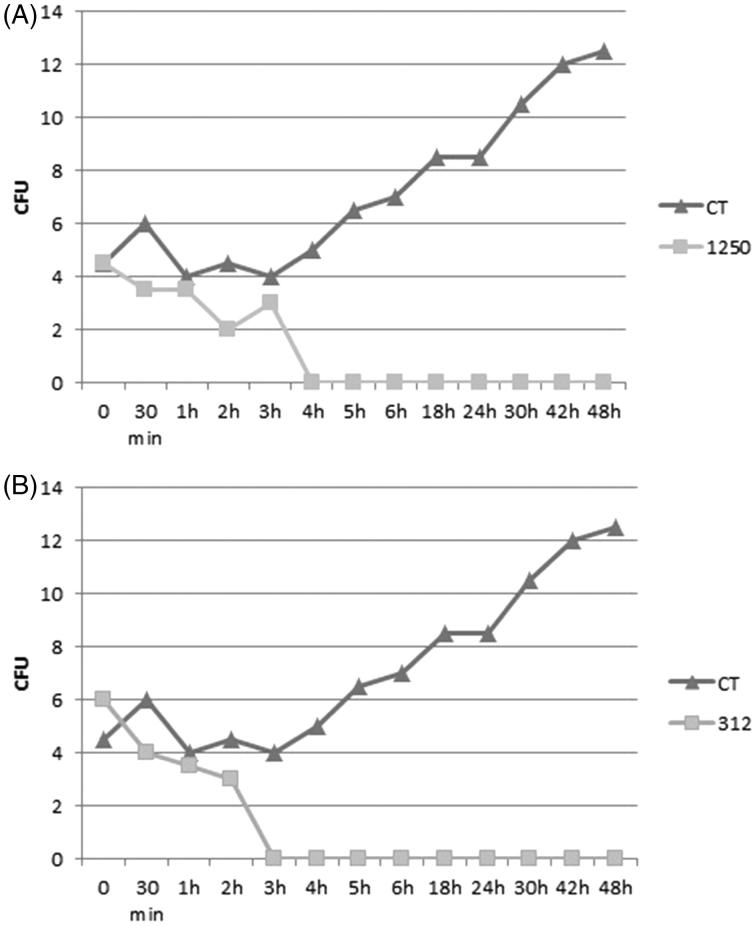
Time-dependent kill-curve assay of 1250 μg/mL E.C.E. (A) and 312 μg/mL H.F. (B) against *C. neoformans* T_444_. E.C.E.: ethanol crude extract; H.F.: hexane fraction; CFU: colony-forming unit.

The MFC of E.C.E. and H.F. leads to 100% loss of fungal viability after 4 h treatment with the E.C.E., and after 3 h with H.F (Figure 4). In the study of Akinpelu et al. (2015), 2 h of treatment with the MIC value of the hexane fraction and ethyl acetate fraction of *Cocos nucifera* killed 100% of *K. pneumoniae* inoculum (Akinpelu et al. 2015).

**Figure 5.**
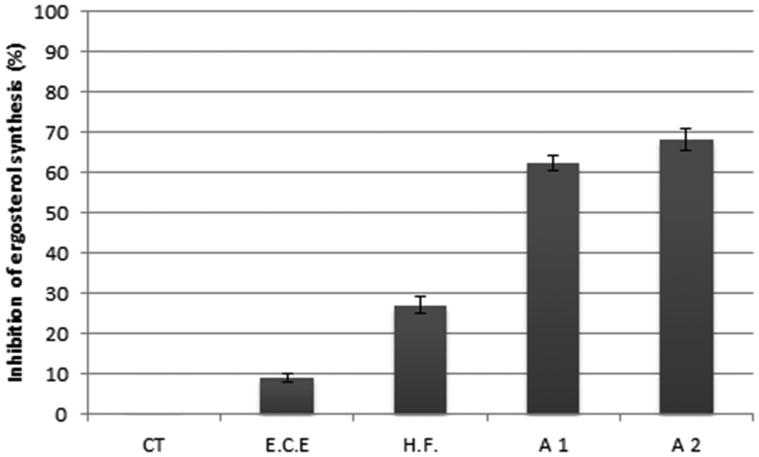
Effect of 312 μg/mL of E.C.E., 78 μg/mL of H.F., 78 μg/mL + 19.5 μg/mL of E.O. and E.C.E., respectively (A1) and 39.36 μg/mL + 10 μg/mL of E.O. and H.F., respectively (A2) on the inhibition of ergosterol synthesis in *C. neoformans*. The results represent the mean ± standard error of two independent experiments in triplicate. A1: association 1; A2: association 2; E.C.E.: ethanol crude extract; H.F.: hexane fraction; E.O.: essential oil.

We also investigate the mechanisms of action of E.C.E., H.F. and their combination with E.O. First, we analyzed the inhibition of ergosterol synthesis. Ergosterol is an important sterol of the yeast cell membrane that controls membrane fluidity and integrity, and it is an important target for some antifungals (White et al. 1998; Andriole 1999). The treatment of *C. neoformans* with E.C.E. (312 μg/mL), H.F. (78 μg/mL), E.C.E. (19.5 μg/mL) + E.O. (78 μg/mL) and H.F. (10 μg/mL) + E.O (39.36 μg/mL) results in an inhibition of 9.09, 27.27, 62.46 and 68.28% of ergosterol synthesis, respectively (Figure 5). Even in very low concentrations, the substances tested were able to reduce the ergosterol content. This indicated that they probably act in the ergosterol biosynthesis pathway.

The results obtained in the ergosterol assay suggest that the association of E.O. with E.C.E. and the association of E.O. with H.F. may act at the cell membrane level, because there was a considerable synthesis inhibition of 62.46% and 68.28%, respectively. This mechanism of action is similar to the azole mechanism, which also acts by inhibiting ergosterol synthesis. The inhibition of ergosterol synthesis in *C. neoformans* was observed in a previous study by our group, and the treatment with E.O. from *O. basilicum* showed 79% of inhibition when compared to control (Cardoso et al. 2016). Tian et al. (2013) studied the inhibition of ergosterol synthesis of *Aspergillus flavus* by E.O. *Anethun graveolens*. In this study, an inhibition of 79.4% at the highest concentration used was reported (1.0 μL/mL) (Tian et al. 2013).

Treatment with H.F. and E.C.E. alone did not result in a high inhibition of the ergosterol synthesis, just 27.27% and 9.09%, respectively (Figure 5). These results suggest that possibly the inhibition of ergosterol synthesis is not their main mechanism of action.

**Figure 6.**
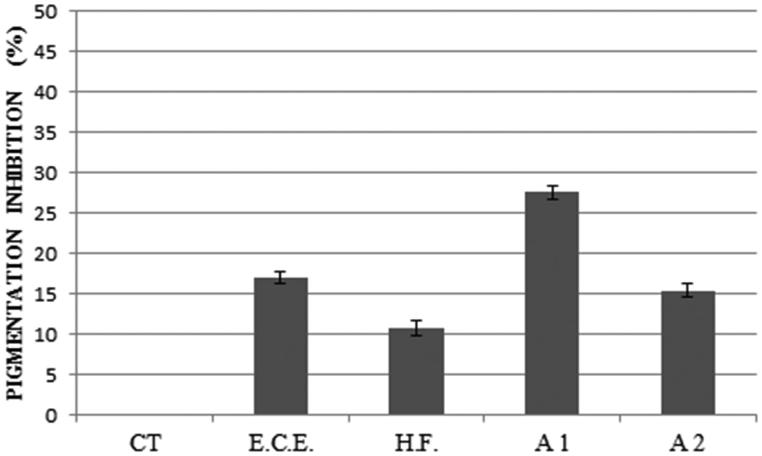
Effect of 312 μg/mL of E.C.E., 78 μg/mL of H.F., 78 μg/mL + 19.5 μg/mL of E.O. and E.C.E., respectively (A1) and 39.36 μg/mL + 10 μg/mL of E.O. and H.F., respectively (A2) on pigmentation inhibition. A1: association 1; A2: association 2; E.C.E.: ethanol crude extract; H.F.: hexane fraction; E.O.: essential oil.

We also evaluated the potential inhibition of E.C.E., H.F. and their combinations on melanin production by *C. neoformans*. A reduction in melanin production was observed in all treatments. Association 1 (E.C.E. + E.O.) showed the best result, followed by E.C.E. alone, Association 2 (H.F. + E.O.) and H.F. alone, respectively (Figure 6).

**Figure 7.**
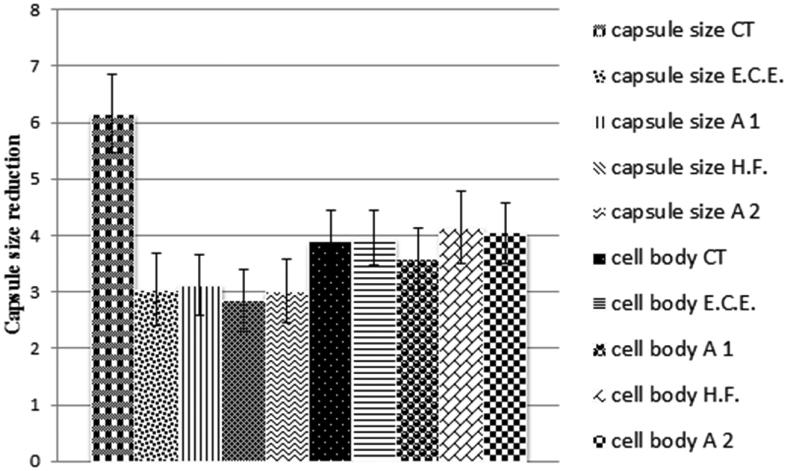
Effect of 312 μg/mL of E.C.E., 78 μg/mL of H.F., 78 μg/mL + 19.5 μg/mL of E.O. and E.C.E., respectively (A1) and 39.36 μg/mL + 10 μg/mL of E.O. and H.F., respectively (A2) on the capsule size (μm). A1: association 1; A2: association 2; E.C.E.: ethanol crude extract; H.F.: hexane fraction; E.O.: essential oil.

Another mechanism of action studied was the ability of the studied components to reduce the capsule size of *C. neoformans*. The discovery of natural products that reduce capsule size is extremely important since the capsule is one of the major virulence factors of *C. neoformans* and can decrease the susceptibility of antimicrobial drugs. The capsule therefore is an important target of study (Zaragoza et al. 2010). In the sub-inhibitory concentrations tested, H.F. showed the highest activity in reducing the capsule size, followed by H.F. + E.O. and E.C.E. E.C.E. + E.O. association was the least active (Figure 7). This result corroborates with those found in the treatment of *C. neoformans* with *O. basilicum* E.O. that showed a capsule size reduction of 16.94% (Cardoso et al. 2016). The results obtained here suggest that the four conditions tested can reduce the capsule size and therefore they might be used with other antimicrobial drugs to reduce the concentration needed and consequently its collateral effects.

The cytotoxic activities of E.C.E. and H.F. were analyzed on a RAW cell line and showed CC_50_ at a concentration of 247 and 90 μg/mL, respectively. Using the checkerboard technique, the MIC value of E.C.E. was reduced from 625 to 78 in combination with AMB and from 625 to 39 μg/mL in combination with E.O. The MIC value of H.F. was reduced from 156 to 78 in combination with AMB and from 156 to 20 μg/mL in combination with E.O. According to this, the MIC values found for both compounds in the associations with AMB and E.O. were much lower than the CC_50_, which minimizes the cytotoxic activity of this component.

## Conclusions

In this study we showed that the hexane fraction has the highest activity against *C. neoformans* T_444_, but E.C.E. presents the lowest cytotoxicity. The ergosterol and pigmentation inhibition, the capsule size reduction and the synergism showed the potential activity of this natural product and that its combination with standard drugs or another natural product can be useful against the microorganisms tested.

Moreover, the antifungal activity found in this study supports the use of *O. basilicum* ethanol extract as folk medicine and further investigations should be made to develop new antifungal agents with effective substances from vegetal origin.
